# The practice of radiation protection in an interventional neuroradiology service

**DOI:** 10.47626/1679-4435-2022-748

**Published:** 2023-02-03

**Authors:** Luciana Machado Sebastião, Rita de Cássia Flôr, Tiago Jorge Anderson

**Affiliations:** 1 Radiology and Imaging Sector, Hospital Governador Celso Ramos, Florianópolis, SC, Brazil; 2 Professional Master’s Program in Radiological Protection, Instituto Federal de Santa Catarina, Florianópolis, SC, Brazil; 3 Hospital Universitário Polydoro Ernani de São Thiago, Universidade Federal de Santa Catarina, Florianópolis, SC, Brazil

**Keywords:** radiology, interventional, radiation protection, occupational health., radiologia intervencionista, proteção radiológica, saúde do trabalhador.

## Abstract

**Introduction:**

Interventional neuroradiology procedures subject professionals who work in this area to high doses of ionizing radiation, and such exposure leads to a higher chance of occupational diseases related to this physical risk. Radiation protection practices aim to reduce the occurrence of such damage to the health of these workers.

**Objectives:**

To identify how the practice of radiation protection occurs in a multidisciplinary team of an interventional neuroradiology service in the state of Santa Catarina, Brazil.

**Methods:**

A qualitative, exploratory, and descriptive research conducted with nine health professionals from the multidisciplinary team. Non-participant observation and a survey form were used as data collection techniques. For data analysis, descriptive analysis based on absolute and relative frequency and content analysis were used.

**Results:**

Although some practices showed the use of radiation protection measures in practice, such as workers taking turns to perform procedures and continuous use of the lead apron as well as the mobile suspended protection, we found that most of the practices violate the principles of radiation protection. Among these inadequate radiological protection practices, the following aspects were observed: not wearing lead goggles, not using collimation to obtain the image, poor knowledge of the principles of radiation protection and biological effects of ionizing radiation, and non-use of an individual dosimeter.

**Conclusions:**

There was a lack of know-how of the multidisciplinary team working in interventional neuroradiology regarding the practice of radiation protection.

## Abstract (pt)

**Introdução:**

Os procedimentos de neurorradiologia intervencionista submetem os profissionais que atuam nessa área a altas doses de radiação ionizante, sendo que tal exposição leva a uma maior probabilidade de ocorrência de doenças ocupacionais relacionadas a esse risco físico. As práticas de proteção radiológica visam a reduzir a ocorrência desses danos à saúde desses trabalhadores.

**Objetivos:**

Identificar como ocorre a prática de proteção radiológica de uma equipe multiprofissional de um serviço de neurorradiologia intervencionista do estado de Santa Catarina, Brasil.

**Métodos:**

Pesquisa qualitativa, exploratória, descritiva, realizada com nove profissionais da saúde da equipe multiprofissional. Utilizou-se como técnica de coleta de dados a observação não participante e um questionário. Para a análise dos dados, empregaram-se a análise descritiva embasada na frequência absoluta e relativa e a análise de conteúdo.

**Resultados:**

Apesar de algumas práticas demonstrarem aplicação de medidas de proteção radiológica na prática, como revezamento de trabalhadores para execução de procedimentos e uso contínuo do avental plumbífero, bem como do anteparo móvel suspenso, verificaram-se, em sua grande maioria, práticas que ferem os princípios de proteção radiológica. Entre essas práticas de proteção radiológica não atendidas, foram observados os seguintes aspectos: não uso de óculos plumbíferos, não emprego de colimação para aquisição de imagem, conhecimento incipiente dos princípios de proteção radiológica e efeitos biológicos da radiação ionizante, não uso de dosímetro individual.

**Conclusões:**

Evidenciou-se um saber-fazer incipiente da equipe multiprofissional que atua em neurorradiologia intervencionista quanto à prática de proteção radiológica.

## INTRODUCTION

Interventional radiology applied to angioradiology is an area of medical practice that covers diagnostic and therapeutic interventions guided by different imaging methods, including fluoroscopy and angiography. Minimally invasive techniques are used in association with imaging methods to locate and treat pathologies.^^1^^ Neuroradiology aims to treat pathologies such as strokes, brain tumors, aneurysms, and other central nervous system complications using endovascular approaches.^^2^^

It is a fact that interventional neuroradiology procedures subject professionals working in this area to high doses of ionizing radiation due to the prolonged use of fluoroscopy and repetitive acquisition of angiography images.^^3^,^4^^ Such occupational exposure presents a higher probability of cancer occurrence.

The relationship between occupational exposure to ionizing radiation and the increased cancer incidence has been evidenced since the early days of the use of ionizing radiation in diagnostic and therapeutic interventions, and the occurrence of cancer is a significant concern among professionals working in interventional radiology.^^5^^ Among the pathologies associated with the performance of neuroradiological procedures are radiodermatitis and radiogenic cataracts.^^6^^

Therefore, there is a need to implement an effective program of radiological protection in interventional radiology, which must contemplate items such as exposure monitoring strategies, use of radiological protection clothing, time, distance, and continuing education of workers.^^7^^

However, it has been observed that the instruction on radiological protection is incipient among the professionals who work in interventional radiology, resulting in the adoption of erroneous methods regarding the safe use of ionizing radiation in medical practice.^^8^^ Additionally, there is also a low adherence of workers to the use of radiological protection garments, besides the lack of knowledge on the biological effects of ionizing radiation.^^9^,^10^^

It is important to understand how the work process of the professionals who work in interventional neuroradiology is developed to show how the principles of radiological protection are applied in the daily work of these professionals.

This study aimed to identify how the practice of radiological protection occurs in a multidisciplinary team of an interventional neuroradiology service in the state of Santa Catarina, Brazil.

## METHODS

This is a qualitative, exploratory, and descriptive research study. The study was conducted in a public state hospital in the municipality of Florianópolis, state of Santa Catarina, located in southern Brazil. This hospital is a reference center in acute stroke care, as well as in neurology and neuroradiology. The institution has an imaging center with conventional radiology, computed tomography, magnetic resonance imaging, and interventional radiology services. The latter is the object of investigation in this research.

Nine healthcare professionals participated in the study: three neurosurgeons, one anesthesiologist, one nurse, three nursing technicians, and one radiology technician. For data collection, non-participant observation and a questionnaire with objective and subjective questions were used, during the months from October 2017 to January 2018.

In the non-participant observation stage, a previously planned script was used, which was completed to contemplate aspects such as the use of radiological protection clothing and collective protection equipment, the use of individual dosimeters, radiation protection behavior, and equipment and procedure aspects. Descriptive and reflective notes were recorded in a field diary. The work process was systematically observed in the interventional radiology service during the morning shift. The observations covered the arrival and preparation of the professionals and work environments and the completion of the activities involved in the imaging acquisition, totaling 36 hours of observation.

We also applied a closed-ended questionnaire addressing questions about radiological protection, biological effects of ionizing radiation, access to the monthly dose report, occupational dose limits, the ALARA (As Low As Reasonably Achievable) guiding principle of radiation safety, and the use of individual dosimeter and radiological protection clothing.

For the analysis and interpretation of the data related to the administration of the questionnaire, techniques were used to organize, systematize, and interpret the data, applying descriptive statistics based on absolute and relative frequency. These techniques involved computer resources, generating graphs built in the Excel spreadsheet editor. The data from non-participant observation were analyzed using content analysis, and were sorted into four categories: behavior, radiological protection, operational parameters, and knowledge. Finally, the observation and questionnaire data were discussed together, developing the metadata.

In compliance with the ethical guidelines for scientific studies, the project that preceded this study was registered in the Plataforma Brasil (Brazilian database of records of research with humans) and submitted to the Research Ethics Committee of the institution in which the research was conducted. Approval was given under number 2,289,586.

## RESULTS

### BEHAVIOR OF WORK TEAMS

We verified that the number of professionals of each category involved in the procedures was maintained with little variation. In categories with more than one professional, such as neurosurgeons and nursing technicians, two types of situations were observed. In the case of neurosurgeons, the interventions observed occurred under the responsibility of one or two physicians. Among the nursing technicians, two professionals participated during the procedures, one instrumentalist and one room circulator.

These professionals were concerned about alternating their participation in the field. An example of this is the participation of the room circulator technician since he or she may remain outside the procedure room during most of the intervention time. The same happened with the anesthesiologist, who stayed near the door for patient observation and monitoring.

### RADIOLOGICAL PROTECTION

It was verified that, whenever possible, a greater distance was kept from the patient and the tube emitting ionizing radiation, especially by the nursing technicians. This professional category also demonstrated their knowledge of radiological protection when taking turns to participate. However, no change in behavior was perceived when alternating the C-arm position for oblique (mainly left anterior oblique) and lateral imaging acquisition.

The opening of the exam room door by all professional categories during radiation emission was observed in several situations. We also observed a discontinued use of the individual dosimeter by neurologists, nurses, and nursing technicians, and the radiology technician and anesthesiologist did not use it at any point throughout the period of observation.

It was noteworthy that the multidisciplinary team had the usual practice of using radiological protection garments, such as a lead apron, thyroid shield, and lead glasses, except for one of the neurosurgeons, who did not use the glasses at any point during the observations.

As collective protection equipment, the service provided only a ceiling suspended shield, which was used on all occasions by the neurosurgeons.

### OPERATIONAL PARAMETERS

Concerning the exposure area, we observed that there was alternation in the equipment between the geometric magnification selected by the operator and the automatic one related to the position of the C-arch, with FoV 9 and 7 being the most frequent. It is worth mentioning that no collimation of the exposure field was observed during the procedures. The low fluoroscopy mode remained fixed in all procedures, with a pulse rate of 10 exposures/s, and the most frequently used acquisition mode was 2 frames/s, varying to 7 frames/s and R-DSA when necessary. The triggering of the sound signal of five minutes of exposure was recorded at least once on all observation days. Regarding the positioning of the team in relation to the C-arm, one of the neurosurgeons, when obtaining lateral images, routinely used the emitter tube facing his side, the others faced it to the opposite side of the examination table.

### KNOWLEDGE ON RADIOLOGICAL PROTECTION

The members of the nursing team showed an understanding of their protection, such as the concern with taking turns in the procedures. There was a notice posted on the wall of the control room regarding radiological protection, reminding workers of the obligation to wear protective clothing, the correct way to use the dosimeter, and the individual positioning in the examination room.

**Figure 1:**
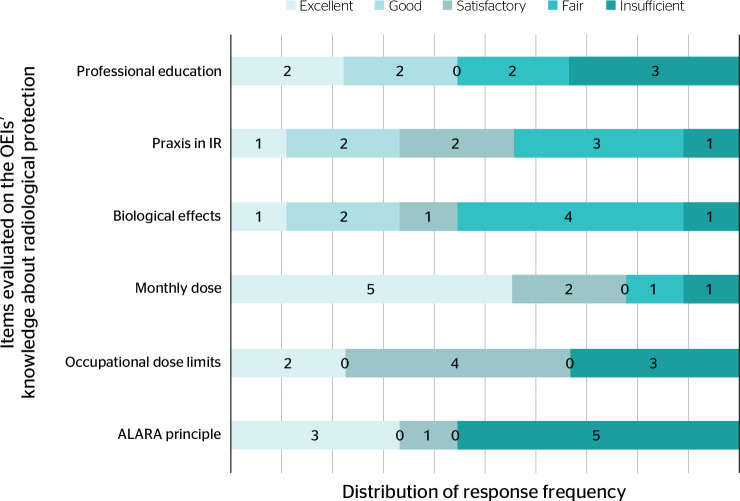
Frequency of responses on occupationally exposed individuals’ knowledge about radiological protection. ALARA = As Low As Reasonably Achievable; OEI = occupationally exposed individual; IR = ionizing radiation.

Regarding the workers’ knowledge, we have the following data as illustrated in Figure 1.

In response to the knowledge acquired during their professional training, four (44%) considered it good or excellent, two (22%) considered it to be fair, and three (33%) insufficient.

Regarding the knowledge related to the practice of ionizing radiation, only one (11%) considered it excellent, four (44%) considered it good or sufficient, three (33%) fair, and one (11%), insufficient.

In relation to the knowledge on biological effects of radiation, only one (11%) considered it to be excellent, two (22%) good, one (11%) satisfactory, four (44%) fair, and one (11%) insufficient.

Concerning knowledge on their radiation dose received monthly, five (55%) said it was excellent, two (22%) had good knowledge, one (11%) considered it fair, and one (11%) considered it insufficient.

Regarding the dose limits established by national legislation, two (22%) reported that they had a very good understanding and full knowledge about the subject; four (44%) classified their knowledge as sufficient, and three (33%) as insufficient.

When asked about the occupational dose limit, two (22%) claimed to have good knowledge, four (44%) claimed it to be satisfactory, and three (33%) insufficient.

Knowledge about the ALARA principle was regarded as very good by three (33%) of the occupationally exposed individuals, sufficient by one (11%), and insufficient by five (55%) participants, that is, more than half of the occupationally exposed individuals were unaware of the principles of radiological protection, corroborating the purpose of this research.

## DISCUSSION

Given the particularities of the work involving exposure to ionizing radiation and, in this context, in an interventional neuroradiology service, it is important for the multiprofessional team to know the basic principles of radiological protection.

When questioned about professional training, 45% of the participants rated their knowledge as good or excellent in radiological protection, as well as the quality and quantity of courses offered by the surveyed institution; however, 55% rated their knowledge during professional training as fair or insufficient. According to these professionals, the institution does not offer the ideal training for their practice, whether in the quantity or in the quality of the content presented. It is important to remember that the International Commission on Radiological Protection (ICRP) 113 suggests that the professionals who have direct involvement with ionizing radiation in their professional attributions should have qualifications and training in radiological protection in their curricula, and the continuity of this process should be maintained throughout their professional career.^^11^^

The knowledge on the specificities of the work process involving exposure to ionizing radiation generally occurs after professional training, either at higher education or technical level, except for those who took courses in radiology, such as the education of professionals in radiological techniques (technologists and technicians in radiology), as well as the medical residency in radiology. For this reason, professionals from other areas who work with ionizing radiation do not always have the necessary technical information on the principles of radiological protection. It was observed that there is a divergence between workers claiming to master the principles of radiological protection and actually applying them in practice.^^12^^ A similar situation was observed among 780 Italian radiologists, of whom only 12.1% reported attending radiological protection courses regularly, and 90% claimed to have sufficient knowledge on radiological protection issues, even though they showed gaps in their knowledge while answering the questions addressing this issue.^^9^^

Even though the role of nursing in procedures involving the use of ionizing radiation is increasing gradually, the training about this work process is also disregarded in the training of nurses and nursing technicians.^^13^^ Although Resolution 211 of the Brazilian Federal Council of Nursing provides on the activities of nursing professionals who work with ionizing radiation has been in force since 1998, it is still observed that when providing care to patients submitted to diagnosis and treatment with ionizing radiation, nurses are not always concerned about their radiological protection.^^14^^

It is also important to highlight that health services assisting to patients utilizing radiological technologies must adopt a permanent education program, as established by the Brazilian legislation for interventional radiology services. Such continuing education programs must contemplate training at the beginning of the activities and periodically, at least once a year, including theoretical and practical training when new processes, techniques, or technologies are implemented or when new people join the working process, as well as the use of an evaluation methodology to demonstrate the effectiveness of the proposed training and capabilities.^^15^^

In the non-participant observation, it was possible to identify the correct behavior of the work teams regarding the basic principles of radiological protection, such as the rotation among workers during the examinations, since this is a way to optimize the exposure to ionizing radiation of the exposed workers.^^16^^

It was also observed that there is the presence of a suspended movable shield as collective protection equipment in the sector, and its use was observed in all procedures. Thus, it is important to highlight that other shielding devices can be used to reduce exposure to ionizing radiation during interventional procedures, such as lead-containing blinds and movable shielding.^^17^^ It is up to the workers to demand the acquisition of this collective protection equipment, and it is up to the institution to acquire it since there is a good adherence of the workers in the use of this equipment.

Inconsistent behaviors concerning these principles were also identified. As an example, one of the medical professionals did not use lead shielding goggles at any point. It is important to emphasize that, for radiological protection purposes, the use of a suspended moving shield, present in the observed scenario, does not exempt the use of goggles by the workers. It is a fact that the use of goggles reduces the radiation exposure dose.^^6^^ In another situation observed, it was found that among 156 physicians who worked with interventional radiology, only 60% of these professionals used protective goggles, justifying the low adherence by the discomfort of the goggles for being heavy and also the difficulty of adaptation to the face. The same study indicated an increase in radiogenic cataracts among exposed workers when compared to the non-exposed group.^^18^^ By respecting the 20 mSv limit for the equivalent lens dose, neuroradiologists, when exposed without the lead-containing goggles, could only perform a maximum of 119 interventional procedures/year, while with the use of such goggles the number would rise to 602 procedures/year.^^19^^ Given the higher incidence of radiogenic cataracts in workers who work in interventional radiology and the possibility of performing a higher number of procedures with the guarantee that the dose threshold will not be exceeded, the use of lead glasses is essential in this environment.

In addition, the professionals also opened the door of the examination room during interventional procedures. This goes against the radiological protection norms, which define that the door of the examination room must be kept closed during interventional procedures.^^10^^

Despite the use of operational parameters favorable to the emission of lower doses of radiation, it was observed the non-use of collimation and activation of the beep after five minutes of continuous use of ionizing radiation. This indicates that there was unnecessary exposure of anatomical structures and prolonged use of the primary radiation beam.

Thus, the need to adjust the image acquisition parameters periodically is highlighted, aiming to use the lowest possible radiation dose. To this end, it is important that neuroradiologists, medical physicists, biomedical engineering, hemodynamic device manufacturers, and other professionals involved in interventional neuroradiology procedures work together.

The search for the improvement of imaging acquisition protocols is something to be pursued in the most diverse interventional radiology settings. Regarding collimation, it should be adjusted to irradiate only the desired area, which results in a decrease in patient and practitioner doses and better image quality. The risk of dose exposure to patients and professionals can be reduced with the use of short fluoroscopy sequences, the use of image freezing, and the use of automatic dose adjustment resources.^^20^^

When asked about the use of the individual dosimeter, seven (78%) of the participants claimed that they always use it correctly, at chest height and over the lead apron. In the observation, however, there was a lot of inconsistency in the use of the dosimeter. Among the nine professionals, only five (55%) were using it routinely, and the anesthesiologist and the radiology technician did not use the dosimeter at any point.

Regarding the monthly radiation dose received, most of them reported knowing it, but they did not use the individual dosimeter that is responsible for recording this dose continuously. Thus, if the dosimeters are used incorrectly or not used by occupationally exposed individuals in interventional radiology services, we may have inconsistency in the record of these doses.^^10^^

When asked about the ALARA principle and the biological effects of radiation, 55% of the professionals indicated that they had fair or insufficient knowledge about the biological effects of radiation. Thus, there was inconsistency between saying that they had satisfactory training, but had little effective knowledge about radiation protection. It is known that ionizing radiation can cause irreversible biological effects to the worker’s health. Prolonged exposure, even to low doses of ionizing radiation, is associated with increased occurrence of leukemia, brain cancer, breast cancer, and melanoma. Many of the side effects generated by continuous exposure to ionizing radiation in medical practice occur after years of exposure.^^20^^ For this reason, the professionals who work in this area, in general, do not relate the occurrence of certain disorders to the effects of exposure to ionizing radiation. This fact was also observed in the present study, in which the professionals reported not having adequate knowledge of the biological effects of ionizing radiation.

## CONCLUSIONS

The practice of radiological protection in interventional neuroradiology is still incipient and requires improvement by workers and management. Although some practices revealed the application of radiological protection measures, such as rotation of workers, continuous use of the lead apron, and the use of the suspended movable shield, most practices violated the principles of radiological protection. Among such practices, it was evidenced the non-use of protective goggles, non-application of collimation during image acquisition, positioning of the X-ray emitting tube towards the hemodynamic equipment operator, incipient knowledge of the ALARA principle and the biological effects of radiation, non-use of the individual dosimeter, and the opening of the examination room door during image acquisition. All these practices violate the principles of radiological protection.

These findings showed a still incipient knowledge of interventional neuroradiology staff regarding the precepts of radiological protection. They also showed that the hospital administration was inefficient in applying radiological protection management. These factors may result in unnecessary exposure of workers and patients to the physical risk of ionizing radiation in the interventional neuroradiology setting.

The small number of participants and the data collection in a single research setting are limitations of the study, which does not allow generalizations about the data found. Future investigations with a larger number of participants and other research designs will provide a greater understanding of the phenomenon, allowing for a more generalized understanding of how the practice of radiological protection occurs in interventional neuroradiology.
